# Hepatitis A virus endemicity and vaccine policy, India

**DOI:** 10.2471/BLT.25.293642

**Published:** 2026-02-20

**Authors:** Avinash R Deoshatwar, Anisha Pulinchani, Basavaraj Mathapati, Madhu Sudan, Suman Manohar Mhatre, Anuradha Patil, Supriya Hundekar, Ganesh Chandra Sahoo, Ashish Kumar, Sahil Sharma, Amrit Virk, Mushtaq Khan Kayamkhani, Suresh Choudhary, Preet Khona, Jagadish Nuchin Nuchin, Gopal Dutt, Prashant Kumar, Ragini Mishra, Laisram Raichandra Singh, Johnson Moirangthem, Mruga Dave, Abhijeet V Jadhav, B Shrinivasa, Vidya Arankalle, Kavita S Lole

**Affiliations:** a Indian Council of Medical Research (ICMR) National Institute of Virology, 130/1 Sus Road, Pune, Maharashtra, MH 411021, India.; b Department of Cellular Biochemistry, University of Gottingen, Gottingen, Germany.; c Department of Virology, ICMR Rajendra Memorial Research Institute of Medical Sciences, Patna, India.; d Department of Community Medicine, Ambedkar State Institute of Medical Sciences, SAS Nagar, India.; e State Health Services, Jaipur, India.; f Integrated Disease Surveillance Project District Health Office, Bhilwara, India.; g Integrated Disease Surveillance Project District Health Office, Gadag, India.; h District Health Office, Gadag, India.; i District Health Office, Reasi, India.; j State Integrated Disease Surveillance Project Office, Patna, India.; k State Health Society Bihar, Patna, India.; l District Health Office, Bishnupur, India.; m District Health Office, Bhavnagar, India.; n ICMR National Institute of One Health Research, Nagpur, India.; o Interactive Research School for Health Affairs, Bharati Vidyapeeth, Pune, India.

## Abstract

**Objective:**

To estimate the age-stratified, hepatitis A virus (HAV) seroprevalence in eight Indian states.

**Methods:**

A cross-sectional seroprevalence survey was conducted in 120 rural and 105 urban population clusters across eight Indian states between 12 December 2022 and 28 November 2023. In each cluster, ten participants were randomly selected from each of the age groups: (i) 2 to 4 years; (ii) 5 to 9 years; (iii) 10 to 14 years; (iv) 15 to 30 years; and (v) > 30 years. Serum samples were tested for anti-HAV antibodies.

**Findings:**

Overall, the HAV seroprevalence in the five age groups was 33.2% (95% confidence interval, CI: 30.4–36.2), 51.9% (95% CI: 49.0–54.8), 69.2% (95% CI: 66.6–71.8), 89.7% (95% CI: 88.6–90.8) and 97.4% (95% CI: 96.9–97.8), respectively. The female-to-male ratio was 1.52 : 1 and the HAV seroprevalence was 73.0% (4940/6768) in females versus 63.2% (2821/4453) in males. Overall, HAV endemicity was found to be high-intermediate in study groups in Gujarat, Jammu, Karnataka, Punjab and Rajasthan, high in Bihar, intermediate in Assam and low-intermediate in rural Manipur. As the overall seroprevalence for all children younger than 15 years was 51.9%, substantially more than 40% were at risk of HAV infection.

**Conclusion:**

Although HAV endemicity varied widely across urban and rural study populations in the eight Indian states, it was generally high-intermediate, providing evidence that HAV endemicity in India has declined in recent years. The study’s findings could help Indian policy-makers decide on HAV vaccination for children.

## Résumé

**Objectif:**

Estimer la séroprévalence du virus de l’hépatite A (VHA) par tranche d’âge dans huit États indiens.

**Méthodes:**

Une enquête transversale de séroprévalence a été menée parmi 120 groupes de population rurale et 105 groupes de population urbaine dans huit États indiens du 12 décembre 2022 au 28 novembre 2023. Au sein de chaque groupe, dix participants ont été sélectionnés au hasard dans chacune des tranches d’âge suivantes: (i) 2 à 4 ans; (ii) 5 à 9 ans; (iii) 10 à 14 ans; (iv) 15 à 30 ans; et (v) > 30 ans. Des échantillons de sérum ont été testés pour détecter la présence d’anticorps anti-VHA.

**Résultats:**

Dans l’ensemble, la séroprévalence du VHA dans les cinq groupes d’âge était de 33,2% (IC (intervalle de confiance) à 95%: 30,4–36,2), 51,9% (IC à 95%: 49,0–54,8), 69,2% (IC à 95%: 66,6–71,8), 89,7% (IC à 95%: 88,6–90,8) et 97,4% (IC à 95%: 96,9–97,8), respectivement. Le rapport femmes/hommes était de 1,52:1 et la séroprévalence du VHA était de 73,0% (4940/6768) chez les femmes contre 63,2% (2821/4453) chez les hommes. Dans l’ensemble, l’endémicité du VHA s’est avérée élevée à moyenne dans les groupes étudiés au Gujarat, au Jammu-et-Cachemire, au Karnataka, au Pendjab et au Rajasthan, élevée au Bihar, moyenne en Assam et faible à moyenne dans les zones rurales du Manipur. La séroprévalence globale chez tous les enfants de moins de 15 ans étant de 51,9%, plus de 40% d’entre eux étaient exposés à un risque d’infection par le VHA.

**Conclusion:**

Bien que l’endémicité du VHA variait considérablement entre les populations urbaines et rurales étudiées dans les huit États indiens, elle était généralement élevée à moyenne, ce qui prouve que l’endémicité du VHA en Inde a diminué ces dernières années. Les résultats de l’étude pourraient aider les décideurs politiques indiens à prendre des décisions concernant la vaccination des enfants contre le VHA.

## Resumen

**Objetivo:**

Estimar la seroprevalencia del virus de la hepatitis A (VHA) estratificada por edad en ocho estados de la India.

**Métodos:**

Se realizó una encuesta transversal de seroprevalencia en 120 conglomerados de población rurales y 105 urbanos en ocho estados de la India entre el 12 de diciembre de 2022 y el 28 de noviembre de 2023. En cada conglomerado, se seleccionaron aleatoriamente diez participantes de cada uno de los siguientes grupos de edad: (i) 2 a 4 años; (ii) 5 a 9 años; (iii) 10 a 14 años; (iv) 15 a 30 años; y (v) >30 años. Las muestras de suero se analizaron para detectar anticuerpos anti-VHA.

**Resultados:**

En general, la seroprevalencia de VHA en los cinco grupos de edad fue del 33,2% (intervalo de confianza del 95% [IC]: 30,4-36,2), 51,9% (IC del 95%: 49,0-54,8), 69,2% (IC del 95%: 66,6-71,8), 89,7% (IC del 95%: 88,6-90,8) y 97,4% (IC del 95%: 96,9-97,8), respectivamente. La razón mujer:hombre fue de 1,52:1 y la seroprevalencia de VHA fue del 73,0% (4940/6768) en mujeres frente al 63,2% (2821/4453) en hombres. En general, se observó que la endemicidad del VHA fue intermedia-alta en los grupos de estudio de Gujarat, Jammu, Karnataka, Punjab y Rajastán, alta en Bihar, intermedia en Assam e intermedia-baja en zonas rurales de Manipur. Dado que la seroprevalencia global en todos los niños menores de 15 años fue del 51,9%, una proporción sustancialmente superior al 40% estaba en riesgo de infección por VHA.

**Conclusión:**

Aunque la endemicidad del VHA varió ampliamente entre las poblaciones urbanas y rurales estudiadas en los ocho estados de la India, en general fue intermedia-alta, lo que aporta evidencia de que la endemicidad del VHA en la India ha disminuido en los últimos años. Los resultados del estudio podrían ayudar a los responsables de la formulación de políticas en la India a decidir sobre la vacunación frente al VHA en la población infantil.

## ملخص

**الغرض:**

تقدير معدل الانتشار المصلي لفيروس التهاب الكبد أ (HAV) حسب الفئة العمرية في ثماني ولايات هندية.

**الطريقة:**

تم إجراء دراسة استقصائية مقطعية لمعدل الانتشار المصلي في 120 مجموعة سكانية ريفية، و 105 مجموعة سكانية حضرية في ثماني ولايات هندية، بين 12 ديسمبر/كانون أول 2022، و28 نوفمبر/تشرين ثاني 2023. في كل مجموعة، تم اختيار عشرة مشاركين عشوائياً من كل فئة عمرية: (1) 2 إلى 4 سنوات؛ و(2) 5 إلى 9 سنوات؛ و(3) 10 إلى 14 سنة؛ و(4) 15 إلى 30 سنة؛ و(5) أكثر من 30 سنة. تم اختبار عينات المصل للكشف عن الأجسام المضادة لفيروس التهاب الكبد أ.

**النتائج:**

بشكل عام، بلغ معدل الانتشار المصلي لفيروس التهاب الكبد أ في المجموعات العمرية الخمس %33.2 (بفاصل ثقة مقداره %95: 30.4 إلى 36.2)، و%51.9 (بفاصل ثقة مقداره %95: 49.0 إلى 54.8)، و%69.2 (بفاصل ثقة مقداره %95: 66.6 إلى 71.8)، و%89.7 (بفاصل ثقة مقداره %95: 88.6 إلى 90.8)، و%97.4 (بفاصل ثقة مقداره %95: 96.9 إلى 97.8)، على الترتيب. كانت نسبة الإناث إلى الذكور 1.52: 1 وبلغت نسبة الانتشار المصلي لفيروس التهاب الكبد أ %73.0 (4940/6768) لدى الإناث في مقابل %63.2 (2821/4453) لدى الذكور. بشكل عام، تم اكتشاف أن استشراء فيروس التهاب الكبد أ كان متوسطًا إلى مرتفعًا في مجموعات الدراسة في غوجارات وجامو وكارناتاكا والبنجاب وراجستان، ومرتفعًا في بيهار، ومتوسطًا في آسام، ومنخفضًا إلى متوسطًا في المناطق الريفية في مانيبور. ونظرًا لأن معدل الانتشار الإجمالي لجميع الأطفال دون سن 15 عامًا كان %51.9، فإن أكثر من %40 كانوا معرضين لخطر الإصابة بفيروس التهاب الكبد أ.

**الاستنتاج:**

على الرغم من أن انتشار فيروس التهاب الكبد أ تباين بشكل كبير بين التجمعات السكانية الحضرية والريفية في الولايات الهندية الثماني، إلا أنه كان مرتفعًا إلى متوسط بشكل عام، مما يوفر دليلًا على أن انتشار فيروس التهاب الكبد أ في الهند في السنوات الأخيرة. قد تساعد نتائج الدراسة واضعي السياسات الهنود في اتخاذ قرار بشأن تطعيم الأطفال ضد فيروس التهاب الكبد أ.

## 摘要

**目的:**

旨在按年龄分层评估印度八个邦的甲型肝炎病毒 (HAV) 血清阳性反应率。

**方法:**

于 2022 年 12 月 12 日至 2023 年 11 月 28 日期间在印度八个邦共选取 120 个农村人口群和 105 个城市人口群开展了一项血清阳性反应率横断面研究。从每个人口群的以下各个年龄组中分别随机挑选了十名参与者：(i) 2 至 4 岁；(ii) 5 至 9 岁；(iii) 10 至 14 岁；(iv) 15 至 30 岁；以及 (v) 30 岁以上。然后对这些参与者的血清样本进行了抗 HAV 抗体检测。

**结果:**

总体而言，五个年龄组的 HAV 血清阳性反应率分别为 33.2%【95% 置信区间 (CI)：30.4-36.2】；51.9%（95% CI：49.0-54.8）；69.2%（95% CI：66.6-71.8）；89.7%（95% CI：88.6-90.8）；以及 97.4%（95% CI：96.9-97.8）。女性与男性之比为 1.52:1，其中女性的 HAV 血清阳性反应率为 73.0% (4,940/6,768)，男性则为 63.2% (2,821/4,453)。通过检测各研究组以确定 HAV 地方性流行水平，我们发现，总体而言，古吉拉特邦、查谟、卡纳塔克邦、旁遮普邦和拉贾斯坦邦属于中高流行区；比哈尔邦属于高流行区；阿萨姆邦属于中等流行区，而曼尼普尔邦农村地区则属于中低流行区。由于所有 15 岁以下孩子的整体血清阳性反应率为 51.9%，所以存在 HAV 感染风险的孩子所占比例远超 40%。

**结论:**

虽然印度八个邦城市和农村研究人群的 HAV 地方性流行水平差异很大，但是总体属于中高水平，这表明近年来印度的 HAV 地方性流行水平有所下降。该研究的结果有利于帮助印度的政策制定者决定该如何为孩子们安排 HAV 疫苗接种。

## Резюме

**Цель:**

Оценить серопревалентность вируса гепатита А (ВГА) со стратификацией по возрасту для восьми штатов Индии.

**Методы:**

Перекрестное сероэпидемиологическое исследование было проведено в 120 сельских и 105 городских кластерах населения в восьми штатах Индии в период с 12 декабря 2022 года по 28 ноября 2023 года. В каждом кластере были случайным образом отобраны по десять участников из каждой возрастной группы: (i) от 2 до 4 лет; (ii) от 5 до 9 лет; (iii) от 10 до 14 лет; (iv) от 15 до 30 лет; (v) > 30 лет. Образцы их сыворотки были протестированы на наличие антител к ВГА.

**Результаты:**

В целом серопревалентность ВГА в пяти возрастных группах составила 33,2% (95%-й доверительный интервал, ДИ: 30,4–36,2), 51,9% (95%-й ДИ: 49,0–54,8), 69,2% (95%-й ДИ: 66,6–71,8), 89,7% (95%-й ДИ: 88,6–90,8) и 97,4% (95%-й ДИ: 96,9–97,8) соответственно. Соотношение женщин и мужчин составляло 1,52:1, и серопревалентность ВГА у женщин составила 73,0% (4940/6768) в сравнении с 63,2% (2821/4453) у мужчин. В целом эндемичность по ВГА была оценена как средне-высокая в исследуемых группах в Гуджарате, Джамму, Карнатаке, Пенджабе и Раджастане, высокая в Бихаре, средняя в Ассаме и средне-низкая в сельских районах Манипура. Так как общая серопревалентность для всех детей младше 15 лет составила 51,9%, значительно больше 40% из них имели риск развития инфекции, вызванной вирусом гепатита А.

**Вывод:**

Хотя эндемичность ВГА значительно варьировалась между городской и сельской популяцией в восьми штатах Индии, в целом ее можно охарактеризовать как средне-высокую, что свидетельствует о снижении эндемичности вируса гепатита А в Индии за последние годы. Результаты исследования могут помочь индийским политикам в принятии решения о вакцинировании детей от ВГА.

## Introduction

Since the 1990s, many low- and middle-income countries have reported declining transmission of the hepatitis A virus (HAV).^1^ The virus’s seroprevalence is inversely proportional to the population’s socioeconomic status and is particularly influenced by water quality, sanitation and hygiene because transmission is largely waterborne in these coutries,^2^ though foodborne outbreaks also occur.^3^ As socioeconomic conditions improve, exposure to HAV decreases, leading to a decline in age-related seroprevalence.^4^^–^^6^ Paradoxically, this decline increases the disease burden: the infection is typically asymptomatic in children younger than 5 years but disease severity rises as age at infection increases.^3^^,^^7^^,^^8^ In fact, the age at midpoint of population immunity (i.e. the youngest age at which 50% of individuals have antibodies to the virus) is considered as a reliable indicator of HAV disease burden in the community.^7^

In recent years, hepatitis A has placed a considerable public health burden on low- and middle-income countries, including India. The Global Burden of Disease initiative estimated that the burden of HAV infection in India in 2013 was over 30 disability-adjusted life-years (DALYs) lost per 100 000 population per year;^9^ in 2021, the burden was over 70 DALYs per 100 000 population per year for children aged 5 to 14 years.^10^ India has one of the highest disease burdens globally, and that burden has been increasing rapidly as indicated by rising HAV outbreaks and hospitalizations.^4^^,^^11^^–^^15^ Weekly outbreak surveillance data collected by the Indian government’s integrated disease surveillance programme recorded a marked increase in HAV outbreaks after 2012, a trend likely to continue as water quality, sanitation and hygiene improves.^12^ In 2019, HAV caused 10–30% of acute hepatitis cases in the country and 5–15% of cases of acute liver failure.^16^

Historically, HAV was highly endemic in India, as reported by many studies performed before 2010: the seroprevalence in 10-year-olds was over 90% and the age at midpoint of population immunity was younger than 5 years.^17^^–^^20^ Moreover, a 2014 study of 4175 healthy young adults from various parts of India documented a seroprevalence of 92.7% (95% confidence interval, CI: 91.8–93.5).^21^ However, a systematic review of studies from South-East Asian countries published between 1980 and 2016 included only 14 articles from India, although it did confirm high HAV endemicity in the country.^6^

In low- and middle-income countries, the transition from high to intermediate endemicity has been characterized by HAV infection outbreaks.^7^ Since 2004, Kerala State in India has reported an increasing number of outbreaks involving severe disease and deaths and, in the past decade, there have been around 300 HAV outbreaks.^12^^,^^22^ A recent study indicates that HAV vaccination would be cost-effective in Kerala.^23^ In addition, many other Indian states, including Himachal Pradesh, Jammu and Kashmir, Maharashtra, Punjab and Uttarakhand, have also reported a rise in outbreaks since 2012.^12^

According to a 2022 World Health Organization (WHO) position paper on hepatitis A vaccines,^7^ the inclusion of HAV vaccination in national programmes in low- and middle-income countries is likely to be cost-effective and is recommended when a country transitions from high to intermediate endemicity. Moreover, a national expert committee recently recommended that HAV vaccination should be introduced into India’s universal immunization programme.^24^

As age-stratified seroprevalence estimates are essential for deciding on vaccination policy,^25^ we conducted a population-based, cross-sectional study of HAV seroprevalence in India with the aim of determining whether populations in different states have experienced a decline from high to intermediate HAV endemicity.

## Methods

### Study design and participants

**Fig. 1:**
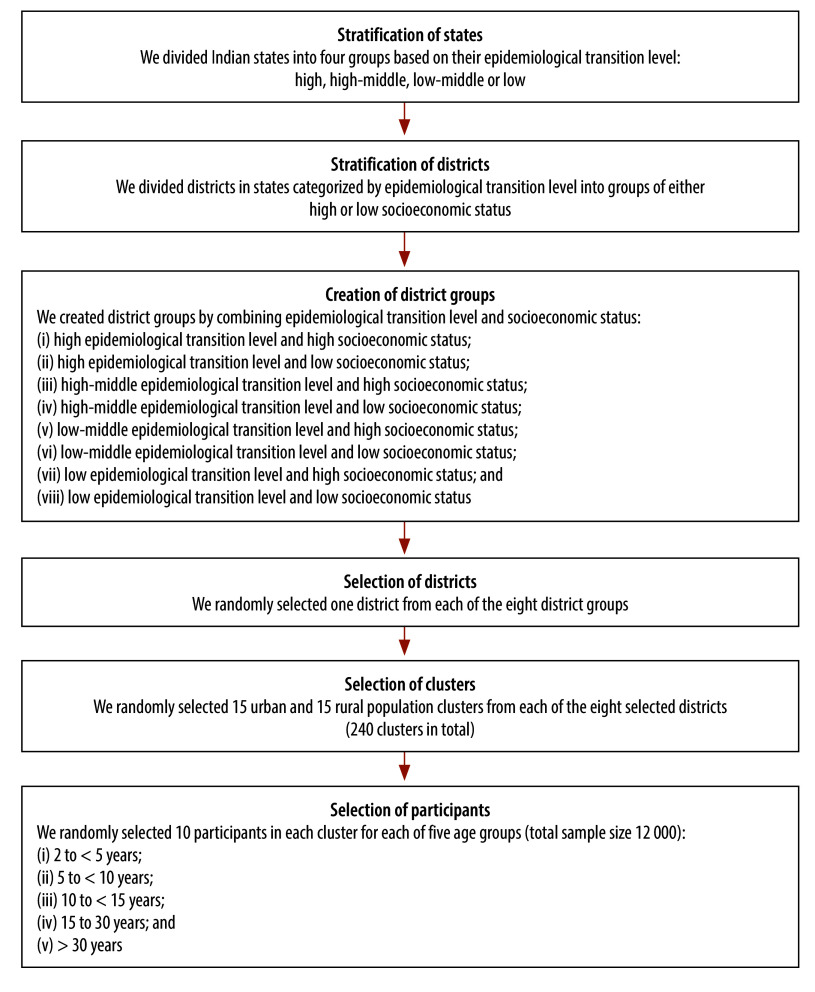
Selection of participants, hepatitis A virus endemicity study, India, 2022–2023

Notes: we defined the epidemiological transition level as the ratio of disability-adjusted life-years lost due to communicable diseases versus noncommunicable diseases in a population. Epidemiological transition levels for 2016 were used for district selection.^26^

Fig. 1 presents a flow diagram of the selection of study participants. Instead of grouping Indian states according to their geographical location, we divided them into groups according to their epidemiological transition level, which is the ratio of DALYs due to communicable diseases versus noncommunicable diseases in a population. Like HAV seroprevalence, the epidemiological transition level is correlated with socioeconomic status.^26^

Participants were recruited in five age groups: (i) 2 to 4 years; (ii) 5 to 9 years; (iii) 10 to 14 years; (iv) 15 to 30 years; and (v) > 30 years. We did not enrol children younger than 2 years because of feasibility and ethical issues, which avoided including infants with maternal antibodies. For the statistical analysis, we considered the seroprevalence in the group aged 2 to 4 years to represent that for all children younger than 5 years.

Based on the findings of a study we conducted in Pune District in 2017,^5^ and assuming 75% seroprevalence, 7.5% precision for the 95% CIs and a design effect of 2, we derived a sample size of 256 participants per age group over 30 clusters, or 8.53 participants per age group in each cluster, using Epi Info v. 7.2.6.0 (Centers for Disease Control and Prevention, Atlanta, United States of America). After adjustment for issues associated with sample quality, transport and handling, we rounded the number up to 10 participants per age group in each cluster, which corresponded to a total of 300 participants per age group over 30 clusters in each district (Fig. 1). Thus, the study was planned to involve eight districts containing 30 clusters of five age groups with 10 participants in each age group, making a total sample size of 12 000. Guidance on vaccination coverage cluster survey design from WHO was used as the reference to validate the sample size calculations.^27^

In the literature,^28^^–^^30^ the endemicity level is assigned according to the age at midpoint of population immunity, as follows: (i) younger than 5 years (high endemicity); (ii) 5 to 10 years (high-intermediate endemicity); (iii) 11 to 15 years (intermediate endemicity); (iv) 16 to 20 years (low-intermediate endemicity); (v) 21 to 30 years (low endemicity); and (vi) older than 30 years (very low endemicity).

We included people living in a particular cluster for more than 6 months in the study. In urban areas, the clusters comprised municipal wards, whereas in rural areas, they were villages. Simple random sampling was used to select clusters because the most recent population data were from the 2011 census and large changes may have occurred since then.

We informed state and district health authorities and local community leaders about the study’s activities and sought their cooperation. A supervisor and coordinator and a team of technicians were selected from study staff and staff at collaborating local institutions. Local health department staff, such as assistant nurse midwives or workers at *Anganwadi* (i.e. rural childcare centres) informed the population about the study at least 1 day before field activities. For villages with a population under 1000, neighbouring villages were included to achieve the desired sample size, particularly for the age groups 2 to 4 years and 5 to 9 years. Within each cluster, the study team established a data and sample collection base at a convenient place for sample collection, preferably near the centre of the location, and villagers residing in households in all compass directions from the base were invited to participate. Initially, we included one family member. However, if not enough families within a cluster were willing to participate, we included more members from willing families until the sample size of 10 participants per age group was reached.

We amended recruitment early in the study because of the surprisingly low HAV seroprevalence we found in districts with a low epidemiological transition level: we replaced one district with a high epidemiological transition level by an additional district with a low epidemiological transition level to reflect the decline in seroprevalence. We could not include the urban population in Manipur because of an eruption of social unrest in the region midway during field activities; however, 762 individuals in rural areas were enrolled before the unrest manifested.

### Data and blood sample collection

After giving informed consent, the participant or parent (if the participant was younger than 18 years) filled in a questionnaire on risk factors. Sex of the participant was recorded as reported by them or parents. In addition, we collected venous blood samples: 3 to 5 mL from individuals older than 5 years and 1 to 2 mL from younger children. The samples were stored in containers at 4 °C 30 minutes after drawing. Following separation, serum samples were stored at 4 °C until couriered to the Indian Council of Medical Research National Institute of Virology on dry ice for testing. Samples were tested for the presence of anti-HAV antibodies using the HAV Ab competitive enzyme-linked immunosorbent assay (DIA.PRO, Milan, Italy). The sensitivity and specificity reported by the manufacturer were 100% and over  98%, respectively.

### Statistical analysis

We calculated overall age- and gender-weighted seropositivity rates for various sociodemographic and hygiene variables across study districts categorized by socioeconomic status. In addition, we also determined the overall seroprevalence for each age group, for urban and rural areas and for both sexes in total and for individual study districts. To derive weighting, we used age and sex distributions for India from the 2021 National Health Profile.^31^ To determine age at the midpoint of population immunity, we created smaller 3-year age groups (e.g. 2 to 4 years, 5 to 7 years and 8 to 10 years) and plotted the seroprevalence in these groups. The statistical analysis was performed using R v. 4.3.1 (The R Foundation, Vienna, Austria).

The study was approved by the institutional ethics committee of the Indian Council of Medical Research National Institute of Virology in January 2020. Written informed consent was obtained from participants 18 years or older, parental consent was obtained for younger participants, and children aged between 7 and 17 years gave assent.

## Results

Between 12 December 2022 and 28 November 2023, a total of 11 380 individuals were recruited from eight districts across eight states: Bhilwara (Rajasthan), Bhavnagar (Gujarat), Patna (Bihar), Bishnupur (Manipur), Dibrugarh (Assam), Reasi (Jammu), Gadag (Karnataka) and Sahibzada Ajit Singh Nagar (Punjab).

Blood samples from 11 231 individuals were of sufficient quantity and quality; 54 individuals refused to give samples. The planned sample size of 12 000 was not reached because of social unrest in Manipur.

Table 1 shows the demographic details of the participants; 6050 (53.9%) were from rural areas, 4586 (41.3%) reported a family size greater than five, 7633 (68.0%) of mothers had no or only primary school education and the female-to-male ratio was 1.52 : 1 (6768 : 4463). The higher enrolment of females was probably due to the timing of field work, usually between 9:00 am and 4:00 pm. Details of analyses by gender and urban or rural location are available from the corresponding author on request.

**Table 1:** Sociodemographic and hygiene characteristics of participants, study of hepatitis A virus endemicity, India, 2022–2023

Characteristic	No. participants (%) (*n* = 11 231)
**Age group, years**	
2 to 4	2 080 (18.5)
5 to 9	2 273 (20.2)
10 to 14	2 233 (19.9)
15 to 30	2 344 (20.9)
Over 30	2 301 (20.5)
**Sex**	
Female	6 768 (60.3)
Male	4 463 (39.7)
**Residence location**	
Rural	6 050 (53.9)
Urban	5 181 (46.1)
**Mother’s educational level**	
No formal education	6 031 (53.8)
Primary school	1 602 (14.3)
Secondary school	627 (5.6)
High school	2 569 (22.9)
Undergraduate school	215 (1.9)
Graduate school	157 (1.4)
Data missing	30 (NA)
**Family size**	
≤ 5	6 524 (58.7)
> 5	4 586 (41.3)
Data missing	121 (NA)
**House type**	
Brick or concrete (i.e. *pucca*) house	6 991 (62.5)
House made from natural materials (i.e. *kutcha* house)	4 186 (37.5)
Data missing	54 (NA)
**Drinking water supply**	
Government or municipal supply to tap	6 951 (62.2)
Common community source	2 141 (19.2)
Well	2 088 (18.7)
Data missing	51 (NA)
**Drinking water processing**	
None	9 394 (84.0)
Reverse osmosis filter	1 013 (9.1)
Boiling	774 (6.9)
Data missing	50 (NA)
**Toilet available**	
Yes	9 764 (87.3)
No	1 417 (12.7)
Data missing	50 (NA)
**Reported handwashing with soap after defecation**	
Yes	10 244 (91.6)
No	937 (8.4)
Data missing	50 (NA)
**Reported handwashing with soap before eating**	
Yes	9 995 (89.4)
No	1 186 (10.6)
Data missing	50 (NA)

NA: not applicable.

Notes: inconsistencies arise in some values due to rounding. Data missing values not included in the percentage calculation.

The HAV seroprevalence in the study population for different socioeconomic and hygiene variables is reported in Table 2 for districts of low and high socioeconomic status, respectively. The overall age- and gender-weighted seroprevalence was 81.4% (95% CI: 80.6–82.1). For urban and rural areas combined, the gender-weighted seroprevalence was 33.2% (95% CI: 30.4–36.2) in participants aged 2 to 4 years; 51.9% (95% CI: 49.0–54.8) in those aged 5 to 9 years; 69.2% (95% CI: 66.6–71.8) in those aged 10 to 14 years; 89.7% (95% CI: 88.6–90.8) in those aged 15 to 30 years; and 97.4% (95% CI: 96.9–97.8) in those older than 30 years. In addition, the seroprevalence was 73.0% (4940/6768) in females and 63.4% (2821/4453) in males. Notably, we found that the seroprevalence in all age groups under 15 years was 51.9% (3416/6586).

**Table 2:** Hepatitis A virus seroprevalence, by district socioeconomic status and participants’ sociodemographic and hygiene characteristics, India, 2022–2023

Characteristic	Participants in districts with a high socioeconomic status (*n* = 4530)				Participants in districts with a low socioeconomic status (*n* = 6701)		
	No. tested for anti-HAV antibodies in blood	No. who tested positive for anti-HAV antibodies in blood	HAV seroprevalence, % (95% CI)		No. tested for anti-HAV antibodies in blood	No. who tested positive for anti-HAV antibodies in blood	HAV seroprevalence, % (95% CI)
**Age group, years**							
2 to 4	1025	428	41.8 (38.7–44.8)		1432	404	28.2 (25.9–30.6)
5 to 9	725	472	65.1 (61.5–68.5)		1171	567	48.4 (45.5–51.3)
10 to 14	898	691	76.9 (74.0–79.6)		1335	854	64.0 (61.3–66.5)
15 to 30	949	900	94.8 (93.2–96.1)		1395	1203	86.2 (84.3–88.0)
Over 30	933	920	98.6 (97.6–99.2)		1368	1322	96.6 (95.5–97.5)
**Sex**							
Female	2812	2200	78.2 (76.6–79.7)		3956	2740	69.3 (67.8–70.7)
Male	1718	1211	70.5 (68.3–72.6)		2745	1610	58.7 (56.8–60.5)
**Residence location**							
Rural	2281	1653	72.5 (70.6–74.3)		3769	2433	64.5 (59.8–68.2)
Urban	2249	1758	78.2 (76.4–79.8)		2932	1917	65.4 (63.6–67.1)
**Mother’s educational level**							
No formal education or primary school	3408	2703	79.3 (77.9–80.6)		4225	3131	74.1 (72.7–75.4)
Higher than primary school	1114	702	63.0 (60.1–65.8)		2454	1202	49.0 (47.0–51.0)
**Family size**							
≤ 5	2336	1726	73.9 (72.0–75.6)		4188	2674	63.8 (62.4–65.3)
> 5	2113	1619	76.6 (74.7–78.4)		2473	1642	66.4 (64.5–68.2)
Missing data	81	66	81.5 (71.0–88.9)		40	34	85.0 (69.5–93.7)
**House type**							
Brick or concrete (i.e. *pucca*) house	1098	874	79.6 (77.7–81.9)		3088	1866	60.4 (58.7–62.1)
House made from natural materials (i.e. *kutcha* house)	3378	2494	73.8 (72.3–75.3)		3613	2484	68.8 (67.2–70.3)
**Drinking water supply**							
Common community source	537	473	88.1 (85.0–90.6)		1604	976	60.8 (58.4–63.2)
Government or municipal supply to tap	3310	2410	72.8 (71.3–74.3)		3641	2451	67.3 (65.8–68.8)
Well	632	485	76.7 (73.2–79.9)		1456	923	63.4 (60.8–65.9)
**Drinking water processing**							
Boiling	106	78	73.6 (64.0–81.5)		668	346	51.8 (47.9–55.6)
None	4042	3024	74.8 (73.4–76.1)		5352	3589	67.1 (65.8–68.3)
Reverse osmosis filter	332	267	80.4 (75.6–84.5)		681	415	60.9 (57.1–64.6)
**Toilet available**							
No	458	403	88.0 (84.6–90.7)		959	674	70.3 (67.3–73.1)
Yes	4022	2966	73.7 (72.3–75.1)		5741	3676	64.0 (62.8–65.3)
**Handwashing with soap after defecation**							
No	322	291	90.4 (86.5–93.3)		615	407	66.2 (59.5–67.1)
Yes	4158	3078	74.0 (72.7–75.3)		6086	3943	64.8 (63.5–65.9)
**Handwashing with soap before eating**							
No	544	474	87.1 (84.0–89.8)		642	418	65.1 (61.3–68.8)
Yes	3936	2895	73.5 (72.1–74.9)		6059	3932	64.9 (63.7–66.1)

CI: confidence interval; HAV: hepatitis A virus.

**Fig. 2:**
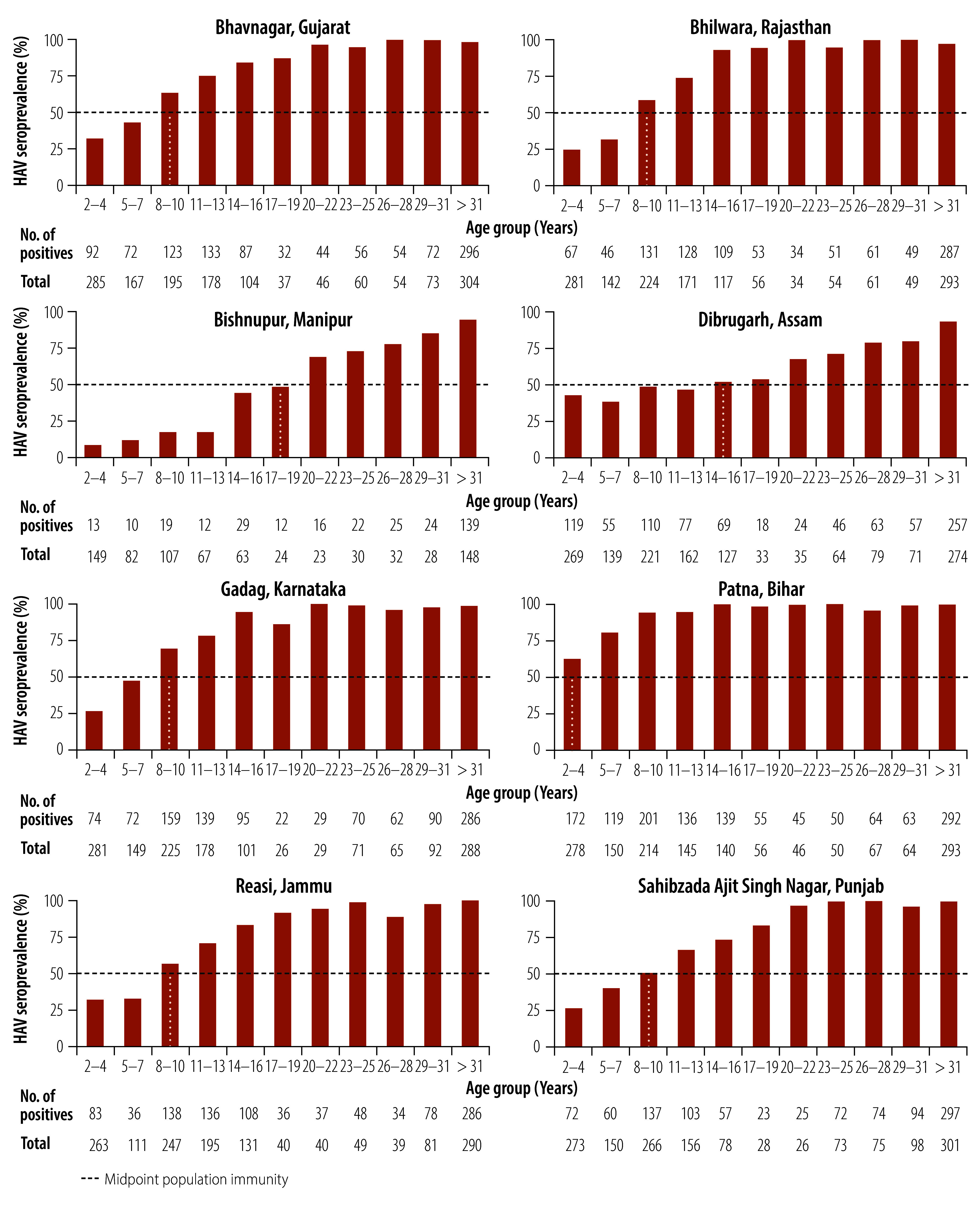
Hepatitis A virus seroprevalence, by study district and age group, India, 2022–2023

Fig. 2 shows the overall seroprevalence by age group in the eight study districts for urban and rural areas combined. Interestingly, though the district of Bishnupur in Manipur had a low socioeconomic status, the estimated HAV seroprevalence was only 15.5% (95% CI: 12.1–18.9) among rural children aged 2 to 14 years, the lowest among study districts for that age group.

HAV: hepatitis A virus.

Notes: the graphs show the seroprevalence for urban and rural areas combined. The dashed line shows the 50% HAV seroprevalence level, which is used to determine age at midpoint of population immunity, an indicator of the level of HAV endemicity. Bars with dotted lines shows the age group for which the midpoint has been passed.

The mother’s educational level was strongly associated with the estimated HAV seroprevalence (*χ^2^* for trend: 735.2; *P* < 0.1). The seroprevalence was 79.2% (95% CI: 78.2–80.2; 4777/6031) for no formal education; 66.0% (95% CI: 63.6–68.3; 1057/1602) for primary education; 49.6% (95% CI: 45.6–53.6; 311/627) for secondary school education; 55.6% (95% CI: 53.7–57.6; 1429/2569) for high school education; 41.4% (95% CI: 34.7–48.3; 89/215) for undergraduate-level education; and 47.8% (95% CI: 39.7–55.9; 75/157) for graduate-level education. Seroprevalence was lower in rural population groups than urban population groups, which mostly had a lower socioeconomic status: 67.5% (95% CI: 66.4–68.7; 4086/6050) versus 70.9% (95% CI: 69.7–72.2; 3675/5181), respectively.

Most study participants were from districts with a low socioeconomic status. Overall, 11 281 participants chose to report their annual family income: it was below 250 000 Indian rupees (₹; approximately 3082 United States dollars, US$, in December 2022) for 10 694 participants (94.8%); between ₹250 000 and 600 000 (i.e. US$ 3082 to 7400) for 522 (4.6%); between ₹600 001 and 1 000 000 (i.e. US$ 74010 to 12 330) for 53 (0.5%); and above ₹1 000 000 (i.e. above US$ 12 330) for 12 (0.1%).

Table 3 shows the level of HAV endemicity in rural and urban populations in the study districts. According to the age at midpoint of population immunity, most districts had high-intermediate, intermediate or low-intermediate endemicity. Notably, the age at midpoint of population immunity in rural Dibrugarh in Assam was 3 years but the cumulative seroprevalence at the age of 15 years was 45.7%.

**Table 3:** Level of hepatitis A virus endemicity and age at midpoint of population immunity, by study district, India, 2022–2023

District, state	Type of community	Age at midpoint of population immunity,^a^ years	Level of HAV endemicity^b^
Bhilwara, Rajasthan	Urban	9	High-intermediate
	Rural	9	High-intermediate
Bhavnagar, Gujarat	Urban	6	High-intermediate
	Rural	9	High-intermediate
Gadag, Karnataka	Urban	9	High-intermediate
	Rural	6	High-intermediate
Patna, Bihar	Urban	3	High
	Rural	3	High
Dibrugarh, Assam	Urban	15	Intermediate
	Rural	3	High
Sahibzada Ajit Singh Nagar (Mohali), Punjab	Urban	9	High-intermediate
	Rural	12	Intermediate
Reasi, Jammu	Urban	9	High-intermediate
	Rural	12	Intermediate
Bishnupur, Manipur	Urban	ND^c^	ND^c^
	Rural	18	Low-intermediate

HAV: hepatitis A virus; ND: not determined.

^a^ The age at midpoint of population immunity is defined as the youngest age at which 50% of individuals have HAV antibodies.

^b^ We determined the level of HAV endemicity in a district from the age at midpoint of population immunity, as follows: (i) < 5 years (high); (ii) 5 to 10 years (high-intermediate); (iii) 11 to 15 years (intermediate); (iv) 16 to 20 years (low-intermediate); (v) 21 to 30 years (low); and (vi) > 30 years (very low).

^c^ Data for the urban population in Manipur could not be obtained because of social unrest during field activities.

## Discussion

Over the past three decades, more than 25 low- and middle-income countries have introduced HAV vaccine into their immunization programmes and universal mass vaccination has been reported to affect disease incidence, mortality and outbreaks in low-, middle- and high-income countries.^32^^–^^36^ Both WHO and others have recommended that HAV vaccine be included in national programmes when a country transitions from high to intermediate endemicity.^7^^,^^37^ In India, there are indications that HAV transmission has been declining in recent years.^12^^,^^13^^,^^15^ Our community-based study of urban and rural areas in eight different states provides evidence of that decline.

Research indicates that improvements to water quality, sanitation and hygiene can lead to a decline in HAV transmission.^38^^,^^39^ In India, there has recently been a rise in HAV-related hospitalizations among children and young adults.^40^ Moreover, HAV infection has been associated with worse outcomes in around 7% of chronic liver disease patients.^14^ However, the lack of accurate data on the HAV disease burden in India remains a major challenge.

Our approach of grouping Indian states according to their epidemiological transition level proved to be useful, as evident from our trend analysis. While studying all Indian states classified by their mix of urban and rural areas and their economic status would have been ideal, this approach would have been expensive and required an unfeasibly large study. Nevertheless, our unexpected findings in Manipur indicate that our approach should be used with caution, as this state neither has high economic status nor high epidemiological transition level. The reasons for the low HAV seroprevalence found in Bishnupur District in Manipur need to be investigated further. 

We found that the midpoint of population immunity in some study groups was between 10 and 15 years. Infections in this age range have been reported to be associated with more severe disease and deaths than in younger age groups.^2^ Consequently, the Indian Academy of Paediatrics has recommended HAV vaccination for children.^41^ For over a decade, however, vaccination has occurred mostly in cities among population groups with a high socioeconomic status. None of the children in our study, who mostly came from families with a low socioeconomic status, had received an HAV vaccine.

Our risk factor analysis found a strong inverse correlation between the mother’s educational level and HAV seroprevalence, which highlights the importance of girls’ education for improving the health of population groups with a low socioeconomic status. This observation may be helpful for policy-makers working on strategic social interventions against infectious diseases, which can be complex.^42^ In contrast with a previous study in India,^18^ we found that HAV seroprevalence was higher in females than males in all study groups. Similar findings have been reported by middle-income countries such as Argentina, Chile, Dominican Republic and Mexico.^43^

Among some study populations, HAV seroprevalence was higher in urban than rural areas, which is in line with our recent observations in Pune District, Maharashtra.^5^ According to data from the Indian government,^44^ 17.4% of India’s urban population lived in slums at the time of the 2011 census and the proportion was likely to be considerably higher in 2025. Targeted policies are required for this population. The income distribution of participants in our study differed from that in India’s population as a whole, where over 25% of households had an annual income above ₹250 000 in 2022.^45^ Consequently, our study findings are predominantly for low-income population groups.

As HAV endemicity progresses through several phases, a single-dose HAV vaccine could be included in the national immunization programme in a phased manner; it could be introduced earlier in states where the age at midpoint of population immunity is high. Although the two-dose regime is more effective,^46^ experience in Brazil showed that inclusion of a single-dose vaccine can give satisfactory results.^47^ In addition, modelling in many countries has shown that including an HAV vaccine in childhood vaccination programmes is cost-effective.^32^^–^^35^^,^^37^ It is reassuring that the vaccine induces robust antibody responses for a long time after vaccination.^48^

Transmission of HAV in India is likely to decline sharply in the near future because of improved socioeconomic conditions, better water quality due to the Ministry of Jal Shakti’s *Har Ghar Jal* mission to provide clean tap water to every household, and continuing efforts to build millions of toilets under the *Swachh Bharat* (Clean India) mission.

Our study has a few limitations. First, seroprevalence estimates weighted by probability proportional to population size were unavailable. Second, in calculating the required sample size, we applied the same assumptions for all socioeconomic categories and age groups. In the absence of national data, we assumed an HAV seroprevalence of 75% for the sample size calculations based on findings in our previous study.^5^ Although generalizing seroprevalence estimates across the country is a limitation from a statistical point of view, we believe our estimates are appropriate for broad endemicity classifications, considering the epidemiology of HAV infection. Third, we used epidemiological transition levels based on 2016 data. Since then, states probably experienced further changes in epidemiological transition level, at different rates. Fourth, we used simple random sampling to select districts and clusters because the population data available were from the 2011 census, and different population groups would have experienced different demographic changes. We selected rural, or village, clusters randomly to ensure that smaller villages had an equal chance of inclusion. Fifth, participation in the study was voluntary and people with a lower socioeconomic status were more willing to participate. Sixth, as population groups with a high epidemiological transition level and high socioeconomic status were underrepresented, we may have overestimated seroprevalence. Moreover, the study enrolled more females than males. As HAV seroprevalence was higher in females, the sex imbalance may also have contributed to the study overestimating seroprevalence. Consequently, HAV endemicity in India is probably lower than indicated by our study.

In conclusion, we obtained community-based data on HAV endemicity across a wide range of geographical regions in India, which indicate that endemicity has declined. Our findings can help policy-makers reach an evidence-based decision on whether an HAV vaccine should be included in the national childhood immunization programme.
